# Complex host genetics influence the microbiome in inflammatory bowel disease

**DOI:** 10.1186/s13073-014-0107-1

**Published:** 2014-12-02

**Authors:** Dan Knights, Mark S Silverberg, Rinse K Weersma, Dirk Gevers, Gerard Dijkstra, Hailiang Huang, Andrea D Tyler, Suzanne van Sommeren, Floris Imhann, Joanne M Stempak, Hu Huang, Pajau Vangay, Gabriel A Al-Ghalith, Caitlin Russell, Jenny Sauk, Jo Knight, Mark J Daly, Curtis Huttenhower, Ramnik J Xavier

**Affiliations:** Department of Computer Science and Engineering, University of Minnesota, Minneapolis, Minnesota 55455 USA; Broad Institute of Harvard and MIT, Cambridge, Massachusetts 02142 USA; Center for Computational and Integrative Biology, Massachusetts General Hospital and Harvard Medical School, Boston, Massachusetts 02114 USA; Biotechnology Institute, University of Minnesota, St. Paul, Minnesota 55108 USA; Zane Cohen Centre for Digestive Diseases, Mount Sinai Hospital IBD Group, University of Toronto, Toronto, Ontario M5G 1X5 Canada; Department of Gastroenterology and Hepatology, University Medical Center Groningen, Groningen, 9700RB The Netherlands; Analytic and Translational Genetics Unit, Massachusetts General Hospital, Boston, Massachusetts 02114 USA; Department of Genetics, University Medical Center Groningen, Groningen, 9700RB The Netherlands; Biomedical Informatics and Computational Biology, University of Minnesota, Minneapolis, Minnesota 55455 USA; Division of Gastroenterology, Massachusetts General Hospital and Harvard Medical School, Boston, Massachusetts 02114 USA; Department of Psychiatry, University of Toronto, Toronto, Ontario M5T 1R8 Canada; Department of Medicine, Analytic and Translational Genetics Unit, Massachusetts General Hospital and Harvard Medical School, Boston, Massachusetts 02114 USA; Program in Medical and Population Genetics, Broad Institute of Harvard and MIT, Cambridge, Massachusetts 02142 USA; Biostatistics Department, Harvard School of Public Health, Boston, Massachusetts 02115 USA

## Abstract

**Background:**

Human genetics and host-associated microbial communities have been associated independently with a wide range of chronic diseases. One of the strongest associations in each case is inflammatory bowel disease (IBD), but disease risk cannot be explained fully by either factor individually. Recent findings point to interactions between host genetics and microbial exposures as important contributors to disease risk in IBD. These include evidence of the partial heritability of the gut microbiota and the conferral of gut mucosal inflammation by microbiome transplant even when the dysbiosis was initially genetically derived. Although there have been several tests for association of individual genetic loci with bacterial taxa, there has been no direct comparison of complex genome-microbiome associations in large cohorts of patients with an immunity-related disease.

**Methods:**

We obtained 16S ribosomal RNA (rRNA) gene sequences from intestinal biopsies as well as host genotype via Immunochip in three independent cohorts totaling 474 individuals. We tested for correlation between relative abundance of bacterial taxa and number of minor alleles at known IBD risk loci, including fine mapping of multiple risk alleles in the Nucleotide-binding oligomerization domain-containing protein 2 (*NOD2*) gene exon. We identified host polymorphisms whose associations with bacterial taxa were conserved across two or more cohorts, and we tested related genes for enrichment of host functional pathways.

**Results:**

We identified and confirmed in two cohorts a significant association between *NOD2* risk allele count and increased relative abundance of Enterobacteriaceae, with directionality of the effect conserved in the third cohort. Forty-eight additional IBD-related SNPs have directionality of their associations with bacterial taxa significantly conserved across two or three cohorts, implicating genes enriched for regulation of innate immune response, the JAK-STAT cascade, and other immunity-related pathways.

**Conclusions:**

These results suggest complex interactions between genetically altered host functional pathways and the structure of the microbiome. Our findings demonstrate the ability to uncover novel associations from paired genome-microbiome data, and they suggest a complex link between host genetics and microbial dysbiosis in subjects with IBD across independent cohorts.

**Electronic supplementary material:**

The online version of this article (doi:10.1186/s13073-014-0107-1) contains supplementary material, which is available to authorized users.

## Background

Crohn’s disease (CD) and ulcerative colitis (UC), collectively known as inflammatory bowel disease (IBD), have long been known to have genetic risk factors due to increased prevalence in relatives of affected individuals as well as higher concordance rates for disease among monozygotic versus dizygotic twins. The sequencing of the human genome and subsequent large-cohort genetic studies has revealed a complex set of polymorphisms conferring varying levels of risk. Extensive analyses of these loci revealed that impaired handling of commensal microbes and pathogens is a prominent factor in disease development [1]. For example, genetically driven impaired function of NOD2 in the sensing of bacterial products like lipopolysaccharide may cause an increase in bacteria that produce those products. Involvement of the JAK-STAT pathway in immune responses, and involvement of the IL-23-Th17 pathway in microbial defense mechanisms, are also possible links between impaired immune response and imbalances in bacterial assemblage [1-3]. These genetic findings are in line with separate, independent tests of microbial shifts associated with IBD. Shifts in taxonomic composition and metabolic capabilities of the IBD microbiome are both now beginning to be defined [4-9]. Determining the extent and nature of host genome-microbiome associations in IBD is an important next step in understanding the mechanisms of pathogenesis. Despite the documented independent associations of IBD with heritable host immune deficiencies and with microbial shifts, there has been limited study of the co-association of complex host genetic factors with microbial composition and metabolism in IBD patients or other populations [9-17], and the mechanisms of host-microbiome disease pathways are largely unknown.

Using three independent cohorts comprising 474 adult human subjects with IBD aged 18 to 75 years, we tested known IBD-associated host genetic loci for enrichment of association with gut microbiome taxonomic composition. Cohorts were located near Boston (USA), Toronto (Canada), and Groningen (the Netherlands), with 152, 160, and 162 subjects, respectively. The cohorts contained 62.5%, 14.3%, and 63.5% CD cases with the remainder cases of UC, and 31.5%, 11.3%, and 53.1% biopsies from inflamed sites, respectively (detailed summary statistics by cohort and biopsy location in Figures S1 and S2 in Additional file 1). The Toronto cohort contained 70.6% biopsies from the pre-pouch ileum in subjects with previous ileo-anal pouch surgery; all remaining samples were from the colon and terminal ileum, with 73.0%, 18.1%, and 87.0% from the colon in the three cohorts, respectively. We excluded all subjects that had taken antibiotics within one month prior to sampling. We obtained genotyping with Illumina Immunochip assays [18] and 16S rRNA gene sequences as described previously [19] (SNP prevalence by cohort in Additional file 2). We rarefied bacterial microbiome samples to an even sequencing depth of 2,000 sequences per sample to control for differential sequencing effort across cohorts. This rarefaction depth allows us to observe taxa with relative abundance as low as 0.15% with 95% confidence in each sample (binomial distribution with 2,000 trials and probability 0.0015). We report a pathway-level analysis of complex functional associations between host genetics and overall microbiome composition, as well as a targeted analysis of the association of *NOD2* with specific bacterial taxa.

## Methods

### Ethics and consent

This study was approved by the Partners Human Research Committee, 116 Huntington Avenue, Boston, MA, USA. Patients gave informed consent to participate in the study. This study conformed to the Helsinki Declaration and to local legislation.

### Data collection and generation

We genotyped subjects using the Immunochip platform as described previously [18], excluding polymorphisms with minor allele frequency of 0.1 or below from subsequent testing. 16S rRNA genes were extracted and amplified from intestinal biopsies and sequenced on the Illumina MiSeq platform using published methods [20]. These procedures include extraction using the QIAamp DNA Stool Mini Kit (Qiagen, Inc., Valencia, CA, USA) according to the manufacturer’s instructions with minor alterations described in prior work [20], followed by amplification using the 16S variable region 4 forward primer GTGCCAGCMGCCGCGGTAA and reverse primer GGACTACHVGGGTWTCTAAT, followed by barcoded multiplexing and sequencing. Only one biopsy was used per subject; when multiple biopsies were available we selected the non-inflamed biopsy first.

### Data processing

We extracted risk allele counts for 163 published genetic risk loci for CD, UC, and IBD [1]. When combining data from separate Immunochip runs we tested for strand inversions by linkage disequilibrium with neighboring variants using plink [21]. Microbial operational taxonomic units (OTUs) and their taxonomic assignments were obtained using default settings in QIIME version 1.8 [22] by reference-mapping at 97% similarity against representative sequences of 97% OTU in Greengenes (taxa version 4feb2011; metagenome version 12_10) [23]. We used all default settings in QIIME 1.8 for OTU mapping, and we used the pre-assigned taxonomy for the Greengenes OTU representative sequences. Samples were rarefied to an even sequence depth of 2,000 sequences per sample to control for varied sequencing depth. Taxa were collapsed into clusters with >0.95 Pearson’s correlation to remove redundant signals in the data (Additional file 3). Principal coordinates of between-subject distances were obtained from UniFrac [24] distances of OTUs and Jensen-Shannon and Bray-Curtis distances of KEGG (Kyoto Encyclopedia of Genes and Genomes) module and pathway distributions. Bacterial taxa were arcsine-square-root transformed and bacterial functions were power-transformed ('car' package [25]) to stabilize variance and reduce heteroscedasticity.

### Statistical analysis

Linear association tests were performed only within those taxa with nonzero abundance in at least 75% of subjects. Taxa below that threshold were subjected to logistic regression for presence/absence; no such taxa revealed significant associations after correcting for multiple comparisons. To ensure robustness of tests to outliers, subjects with taxon or functional module relative abundance more than three times the interquartile range from the mean were excluded for tests of that feature. Power analysis was performed using the linear effect size that we observed for Enterobacteriaceae when regressing on *NOD2* risk allele count and controlling linearly for clinical covariates (f^2^ = R^2^/(1 - R^2^) = 0.013; R is the coefficient of multiple correlation). Assuming the need to correct for testing of all 163 IBD loci against 22 dominant taxa (3,586 tests; adjusted significance threshold = 1.39 × 10^-5^), we would need at least 3,729 samples to power the full analysis (R ‘pwr’ package power calculation for a linear model with 19 numerator degrees of freedom). Discrete qualitative covariates were re-coded with dichotomous dummy variables representing each class prior to testing. Association of clinical covariates was performed jointly by multiple linear regression. To overcome redundancy between clinical covariates, we clustered clinical covariates based on their pairwise maximum uncertainty coefficients [26], an information-theoretic measure of their degrees of shared information. Continuous-valued covariates were discretized prior to information-theoretic clustering. Complete-linkage clustering was performed to identify groups of covariates in which each covariate contained at least 50% of the information contained in each other covariate. Network plots were created using the igraph [27] package. For the network plot of non-genetic host factors and *NOD2*, width of edges was determined by the ratio of a given covariate’s linear regression coefficient to the mean of the regressed taxon’s relative abundance. Enrichment of a host functional pathway for association with bacterial taxa was assessed by comparing the observed rank product of all host gene-bacterial taxon association tests for all genes in the pathway with the distribution of rank products of 100,000 size-matched pathways randomly generated from the null Immunochip variants described above. Prior to testing, REACTOME pathways with >75% overlap were binned and the largest constituent pathway chosen as a representative for subsequent tests.

## Results and discussion

### Genotype-microbiome associations conserved across independent cohorts

**Figure 1:**
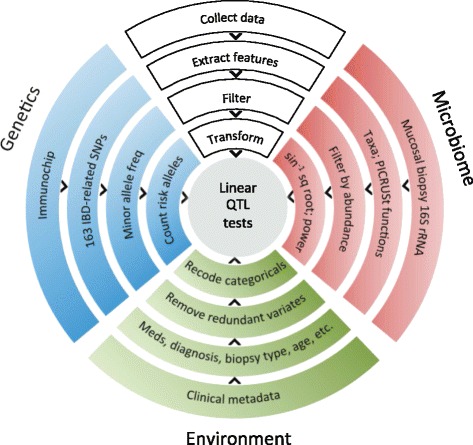
**Schematic of multiomics genotype-microbiome association testing methodology.** Host genome-microbiome association testing involves potentially thousands or millions of genetic polymorphisms and hundreds or thousands of bacterial taxa and genes. Full feature-by-feature association testing is likely to be underpowered in all but the largest cohorts or meta-analyses; therefore, our methodology includes careful feature selection from both data types. Raw genetic polymorphisms were derived from Immunochip data and filtered by known IBD associations from a large-cohort GWAS study [1]. Microbiome sequences were binned by lineage at all taxonomic levels. After data normalization and filtering (see Methods), a simple linear test was performed for association between minor allele count and bacterial taxon relative abundance while controlling for clinical covariates. QTL, quantitative trait loci.

Our genotype-microbiome association testing methodology included steps to overcome power limitations given the very large number of potential comparisons, to incorporate published knowledge of signaling and metabolic pathways in the host genome, and to control for multiple environmental host factors affecting gut microbiome composition (Figure 1). In a targeted analysis of *NOD2*, we also accounted for multiple causal variants in the genetic locus (Supplementary methods in Additional file 1). After data preprocessing and normalization we tested linearly for association of risk allele count in each SNP with the relative abundance of each bacterial taxon. In all tests, we controlled for recent antibiotic usage (<1 month), recent immunosuppressant usage (<1 month), biopsy inflammation status based on pathology, age, gender, biopsy location, CD/UC diagnosis, disease location, elapsed time since diagnosis, cohort membership, and the first three principal components of genotype variation (Figure 1; Figure S3 in Additional file 1). Although the IBD-related SNPs extracted from the Immunochip data were identified previously in European populations, we do not expect this to limit our findings because our cohorts were mostly of European descent. We validated our linear testing methodology by comparing associations in the Boston cohort with those in the other two cohorts, in addition to performing other sensitivity analyses (Supplementary methods in Additional file 1).

We tested 163 recently IBD-associated SNPs for association with bacterial taxonomic profiles; 154 remained after removing those with low minor allele frequencies or with low call rates in our cohorts (Supplementary methods in Additional file 1). Many of these SNPs have unknown mechanisms and are likely only representative of a signal within the surrounding genomic locus. Therefore, when a single gene was associated previously with a SNP, we refer to that SNP by the gene name for convenience. Due to limited statistical power we were unable to perform a full analysis of all possible SNP-taxon associations (Supplementary methods in Additional file 1). However, we were able to test for the robustness of microbiome-wide associations with a given SNP by comparing the directionality of the SNP-taxon coefficients between independent cohorts. For this test we included only those SNP-taxon associations for a given SNP that were nominally significant (*P* < 0.05) in at least one of the studies being compared. We then obtained Matthew’s correlation coefficient (MCC; also known as the phi coefficient) of the signs (positive or negative) of the SNP-taxon coefficients in one study with the signs of corresponding SNP-taxon coefficients in the second study, and corrected these microbiome-wide tests for multiple comparisons (one MCC test per gene) at a false discovery rate (FDR) of 0.25. We chose the FDR of 0.25 for this analysis due to the large number of tests and the fact that we used the significant results mainly to test for enrichment of certain host pathways, rather than to focus on individual associations. We note that it is important to compare only the directionality of SNP-taxon effects between studies, and not the magnitudes of the SNP-taxon regression coefficients, because the magnitude of a coefficient is closely linked to the mean relative abundance of a given taxon. To decrease bias toward a particular taxonomic level of association [28], we performed these tests using bacterial taxa at all taxonomic levels from phylum to genus, collapsing those with redundant signals. In contrast to using OTU clusters, binning by taxonomy allows inherent flexibility in the level of 16S gene sequence identity within each bin in different lineages.

**Figure 2:**
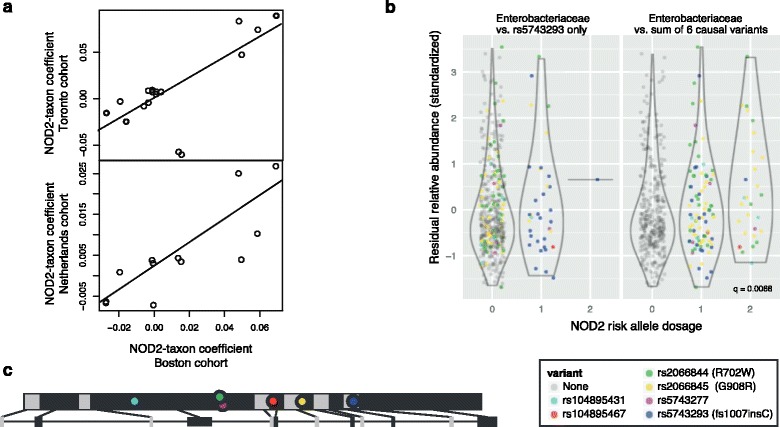
***NOD2*** **fine mapping reveals association with taxonomic and metabolic dysbiosis. (a)** Scatterplot of NOD2-bacterial taxon regression coefficients in one study versus the corresponding regression coefficients in another study. We included only those taxa with a nominally significant (*P* < 0.05) association in a least one of the cohorts being compared. **(b)** Comparison of residual distributions of Enterobacteriaceae with and without incorporating the six independent known causal *NOD2* variants; considering variant rs5743293, only 6.3% of subjects have one or more risk alleles; aggregating risk allele counts across the six variants increases this to 21.8%, and reveals much stronger associations with the microbiome. The strip charts and violin plots show the distribution of standardized residual relative abundance of Enterobacteriaceae versus *NOD2* risk allele dosage after data transformation and regression on clinical covariates. Violin plots show the conditional density of residual relative abundance within each dosage level. **(c)** Relative positions of six *NOD2* variants in *NOD2* exons [29].

A number of host genes, some with known involvement in microbial handling, and others with unknown function, demonstrated reproducible effects on the taxonomic structure of the microbiome across two or more cohorts. Effect size and directionality of genotype-microbiome associations were highly reproducible between cohorts in the case of *NOD2* and 48 other host genes (FDR <0.25; Additional file 4). *NOD2* had one of the most highly reproducible sets of associations with bacterial taxa (MCC = 0.75, FDR = 2.6 × 10^-4^ comparing Boston versus Toronto cohorts; MCC = 0.85, FDR = 7.7 × 10^-4^ Boston versus Netherlands; Figure 2a). Other genes with significantly conserved directionality of effects on bacterial taxa between at least one pair of studies included tumor necrosis factor (ligand) superfamily, member 15 (*TNFSF15*; MCC = 0.87, FDR = 9.5 × 10^-3^, Boston versus Netherlands) and subunit beta of interleukin 12 (*IL12B*; MCC = 0.74, FDR = 1.5 × 10^-3^, Boston versus Netherlands).

*NOD2* variants were the first genetic associations identified in CD, and they remain some of the strongest risk factors. *NOD2*-driven murine dysbiosis causes inflammation even when the dysbiotic microbiota are transplanted into a wild-type mouse [13]. Expression of *TNFSF15*, a member of the tumor necrosis factor ligand superfamily, causes proinflammatory cytokine production, and is specifically expressed more highly in the gut in IBD patients compared with healthy controls. Interestingly, a receptor for a member of the same family, TNFSF14, enhances immune response to pathogenic bacteria via signal transducer and activator of transcription 3 (*STAT3*) activation in a mouse model of *Escherichia coli* infection. TNFSF14 and TNFSF15 are known to share an alternative receptor, indicating potential functional overlap. IL12B forms part of the interleukin-23 complex, involved in microbial defense mechanisms through the IL23-Th17 pathway.

### Immunity-related host functional pathways linked to microbiome profile

**Figure 3:**
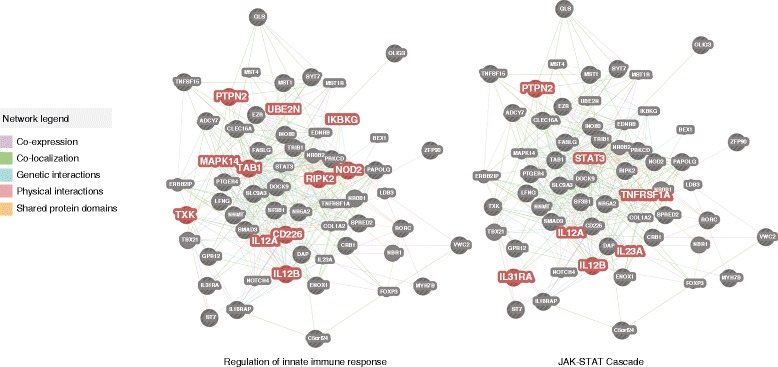
**Host genes with reproducible microbiome associations across cohorts.** Network analysis of host signaling and metabolic pathways enriched for association with microbial taxa (FDR <0.25, Matthew’s correlation test). The visualization of gene-gene interaction network for the subset of 49 genes with significantly conserved directionalities of association with the microbiome is supported by several types of gene-gene connection [30]. This enrichment analysis identified enriched functional networks in innate immune response, inflammatory response, and the JAK-STAT pathway, all of which play roles in immune response to pathogen infection [1].

**Figure 4:**
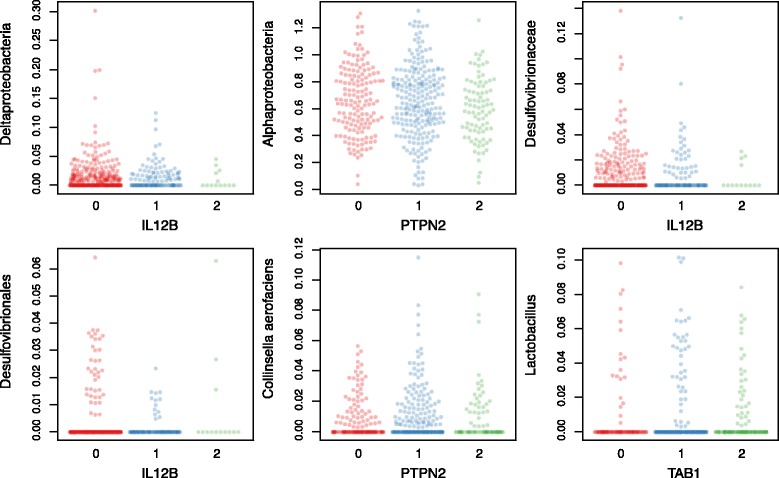
**Top gene-bacteria associations.** Beeswarm plots of the relative abundance of six bacteria stratified by the number of risk alleles present in SNPs in the given genes. The associations shown are the six most significant associations between bacteria and genes in the subset of genes with conserved bacterial associations across cohorts and belonging to the JAK-STAT pathway or the innate immune pathway response as shown in Figure 3. Relative abundances shown are transformed with the arcsine-square root transformation to stabilize variance and to make distributions more normal.

We hypothesized that host functional pathways containing multiple risk variants related to microbial handling and innate immune response would be associated with microbiome features. To test this hypothesis we performed a functional enrichment analysis on the 49 genes identified to have conserved microbiome associations across cohorts. We found these genes to be significantly enriched for regulation of innate immune response (FDR = 2.31 × 10^-6^, hypergeometric enrichment test), inflammatory response (FDR = 7.43 × 10^-6^, hypergeometric enrichment test), and participation in the JAK-STAT cascade (FDR = 2.04 × 10^-4^, hypergeometric enrichment test) (Figures 3 and 4; Additional file 5). A gene-gene interaction network analysis also implicated *STAT3*, interleukin-12 subunit alpha (*IL12A*), and interleukin-23 subunit alpha (*IL23A*) in the network of associated genes.

*STAT3* and *TNFSF15* are both implicated in IL23 signaling. *STAT3* works in concert with Janus Kinase 2 (*JAK2*) in the JAK-STAT pathway to drive immune response to pathogenic infection. *STAT3* also regulates T helper 17 (Th17) cell differentiation by binding IL23 receptor (IL23R; risk variant for IBD: rs11209026) and RAR-related orphan receptor C (RORC; rs4845604), both of which are located in IBD risk loci. *STAT3* defects have also been implicated recently in skin microbial imbalance and impaired host defense. TNFSF15, a member of the tumor necrosis factor ligand superfamily, is a costimulator of T cells, and is specifically expressed more highly in the gut in IBD patients compared with healthy controls [31,32].

### Fine mapping of *NOD2* locus reveals association with Enterobacteriaceae

Based on previous results [9-13] and on the strong linkage between NOD2 and microbial handling [9,12,13], we continued with a targeted analysis of *NOD2* association with specific microbial taxa and functions (Additional file 6). For all *NOD2* testing we aggregated risk allele dosage across six known causal variants: rs2066844 (R702W), rs2066845 (G908R), rs5743277, rs5743293 (fs1007insC), rs104895431 and rs104895467 [29]. This novel methodology was crucial as individual variants contained only part of the signal (Figure 2b,c). *NOD2* was associated with the first principal axis of taxonomic (via weighted UniFrac distances) microbiome variation (FDR <0.05, controlling for three principal axes tested), linking *NOD2* to shifts in overall microbiome taxonomic composition. We identified increased Enterobacteriaceae in subjects with higher *NOD2* risk allele dosage (FDR = 0.11, controlling for multiple taxa tested; Figure 2b; Additional file 7). An increase in Gammaproteobacteria is a known component of IBD dysbiosis and is associated with inflammation in mice and humans [4,33] and with increased epithelial penetration in CD and UC [34]. *NOD2* also had one of the most strongly reproducible associations with microbiome composition when comparing cohorts. Although *NOD2* is only associated with increased risk of CD, *NOD2*-microbiome associations we observed were generally independent of CD/UC diagnosis, with high overlap between CD and UC when tested separately (taxa: Spearman’s rho = 0.57, *P* = 6.5 × 10^-3^; Figure S4 in Additional file 1). This implies that the association may be disease-independent, and may play a role in pathogenesis only in subjects with other risk factors. For example, *NOD2* SNP rs5743293 is associated with complications in ileo-anal pouch patients despite their original diagnosis being UC [35-38].

### A complex web of genotype-environment-microbiome interactions in IBD

**Figure 5:**
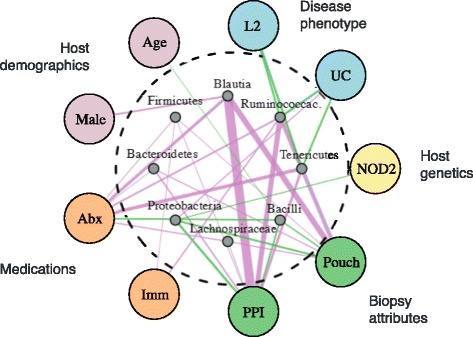
**Host factors associated with the IBD microbiome.** A complex network of host factors associated with the IBD microbiome (all associations FDR <0.05); only taxa with at least four significant associations are included in the network; green and purple edges indicate positive and negative associations, respectively; the width of an edge indicates the strength of the association. The effects of these factors on individual taxa are highly overlapping. The analysis identified covariates representing each type of host factor, consistent with previous results [4]. Biopsy location and medication history had the strongest and most comprehensive effects on microbiome profile; the effect of *NOD2* was moderate in comparison. Cohort membership (not shown) also affected microbiome profile. These results demonstrate the need for study designs and analysis methodologies that control carefully for numerous host genetic and environmental factors when performing microbiome-based biomarker discovery. Abx, antibiotics within 1 month; Imm, immunosuppressants within 1 month; L2, no ileal involvement; PPI, biopsy from pre-pouch ileum.

Our findings indicate that host genetics are part of a complex web of host-associated factors influencing microbiome composition. We performed a meta-analysis of interactions between clinical host factors and bacterial taxa using the above 474 subjects and an additional 55 subjects who had recently taken antibiotics. This analysis included *NOD2* as one of the host factors. We identified an additional 99 significant associations of non-genetic factors with relative abundance of specific bacterial taxa, largely in agreement with previous analyses of the IBD microbial environment [4]. To visualize the overlap of interactions between various host factors and microbial taxa, we constructed a network of associations between bacterial taxa and observed host and environmental factors (Figure 5; Additional file 8; Supplementary methods in Additional file 1).

**Figure 6:**
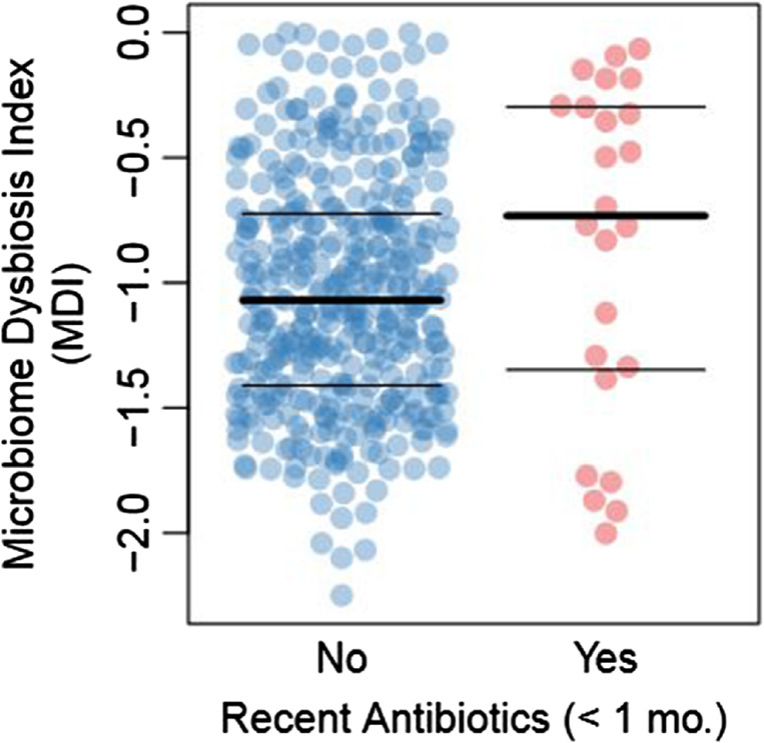
**Association of IBD-related dysbiosis and recent antibiotics usage.** A beeswarm plot [41] of the previously published microbial dysbiosis index [20] (MDI) stratified by recent antibiotics usage by patients. The test for this association between MDI and antibiotics (*P* = 0.039, linear regression *t*-test) included *NOD2* risk allele count to control for the effects of *NOD2* genetics on the microbiome.

This analysis revealed a web of complex overlapping linkages between numerous host factors and bacterial taxa. For example, recent antibiotic usage is associated with systematic shifts in many major taxonomic groups (Figure 6; FDR <0.05); immunosuppressants are associated with decreased Firmicutes, and Ruminococcaceae. Biopsy location and cohort membership had similarly broad effects; age, gender, and disease phenotype had measurable, although less broad, effects; genotype, as represented by the *NOD2* subtype, had a modest effect in relation to other factors. Inflammatory status of the biopsied tissue was associated with increased relative abundance of unclassified members of *Lactobacillus*, and with decreased relative abundance of *Bacteroides uniformis* (Figure S5 in Additional file 1). This analysis demonstrates the comprehensive and intermingled effects of treatment history, gastrointestinal biogeography, and other host and environmental factors on gut microbiome profile and makes clear the need to account for host factors when linking host genotype to microbial composition in a phenotypically heterogeneous population. We confirmed that host genetics as a whole do have a significant effect on microbiome profile by correlating overall between-subject genetic distance (Manhattan distance) with overall between-subject microbiome distance (unweighted UniFrac distance) (*P* < 5.0 × 10^-10^; Figure S6 in Additional file 1), but that it is only a minor contributor in the context of other sources of variation. A recent study of treatment-naïve pediatric patients with CD identified consistent microbiome shifts in patients with recent antibiotic exposure toward the disease-related state [20]. That study exemplified the need to control for the potentially confounding effects of antibiotics when attempting to identify bacterial profiles associated with disease. Based on several studies linking short- and long-term dietary exposure to microbiome profile, it is also likely to be useful to include food intake diaries or dietary recall questionnaires in future genotype-microbiome research [39,40].

### Antibiotics contribute to IBD dysbiosis independent of NOD2 effects

The fact that host genetics are a minor contributor to overall microbiome composition relative to environmental factors does not exclude the possibility that genotype-microbiome interactions play an important role in the etiology of IBD; it is possible that the important variations are in a particular set of taxa or a particular set of functions (for example, resistance to oxidative stress) that make up a minor portion of the overall microbiome, while there are other taxa not closely related to IBD but highly influenced by the host’s environmental exposures (for example, dietary exposures). Such a subset of taxa related to dysbiosis were reported in a recent comparison between treatment-naïve patients with Crohn’s disease and healthy controls [20], and the ratio of the disease-associated taxa to the health-associated taxa was referred to as the microbial dysbiosis index (MDI). This recent study identified an increase in the MDI scores of patients who had recently received antibiotics, indicating that antibiotics tend to shift patient microbiomes further into the realm of IBD-related dysbiosis. We used the same taxa as reported previously to calculate an MDI score for each patient in our analysis. In our cohorts we confirmed the published finding that, when controlling for NOD2 effects on microbiome structure, MDI score tended to be higher in patients with recent usage (within less than one month) of antibiotics (*P* = 0.039, *t*-test of linear regression coefficient) (Figure 6). This finding, together with previously published findings regarding the effects of antibiotics on the IBD microbiome suggest that antibiotics and duration of disease are additional risk factors for IBD-related dysbiosis.

## Conclusions

Taken together, our findings indicate a complex set of associations between the mucosal-adherent microbiome and genetic impairment of several host immune pathways. Although we have been living and evolving with our microbial symbionts throughout human evolution, we have only been aware of their existence for a few centuries, and the genetic and functional diversity of our so-called 'second genome' has only become apparent in the last few decades. Also in recent decades incidence of IBDs and other autoimmune and autoinflammatory diseases has increased dramatically [42], and a rapidly growing set of these diseases has been linked to shifts both in taxonomic carriage and functional potential of host-associated microbial communities. Although our data are cross-sectional and therefore cannot define causality, our analyses demonstrate complex host genetic associations with taxonomic and metabolic dysbiosis in humans. These include implications of microbiome-wide associations with *TNFSF15*, *IL12B*, and with innate immune response, inflammatory response, and the JAK-STAT pathway, as well as *NOD2*-related increases in Enterobacteriaceae relative abundance. Future studies may be warranted to account for the effects of copy number variation, pleiotropic genes and epigenetic modifications. It is also possible that certain genotype-microbiome associations observed in IBD patients may be disease-independent and may be relevant to healthy individuals and individuals with other diseases. The methods we employed were validated on independent cohorts and make possible well-powered false-positive-controlled testing of microbiome-wide host genetic associations.

### Accession numbers

16S rRNA sequences and Immunochip genotyping have been deposited at the National Center for Biotechnology Information as BioProject with top-level umbrella project ID PRJNA205152.

## Additional files

Additional file 1:
**Supplementary methods and figures.**
Additional file 2: Table S1. The prevalence of 163 known IBD-associated SNPs in the three independent cohorts included in this study. Additional file 3: Table S2. Groups of taxa that were binned together prior to statistical testing due to high inter-group correlation. Additional file 4: Table S3. Correlations and significance levels thereof of the directionalities of SNP-taxon associations between pairs of cohorts for those associations that were nominally significant in at least one of the cohorts being compared. Additional file 5: Table S4 Host genetic functional pathways that were significantly enriched for robustness of association with the microbiome across cohorts. Additional file 6:
**Specific SNPs that were included in gene-level fine-mapping of disease association signals.**
Additional file 7:
**Linear association test results for the association of each IBD SNP with each bacterial taxon.**
Additional file 8:
**Additional association test results for a meta-analysis of the three cohorts, including statistics for the associations of clinical covariates with bacterial taxa.**

## Abbreviations

CD: Crohn’s disease
FDR: false discovery rate
IBD: inflammatory bowel disease
IL: interleukin
MCC: Matthew’s correlation coefficient
MDI: microbial dysbiosis index
OTU: operational taxonomic unit
SNP: single nucleotide polymorphism
Th17: T helper 17
UC: ulcerative colitis
