# Supplementary material for: Complex host genetics influence the microbiome in inflammatory bowel disease
Source: Genome Med. 2014 Dec 2;6(12):107. doi: 10.1186/s13073-014-0107-1 (PMC4292994; doi:10.1186/s13073-014-0107-1)
Supplement: Additional file 1: — Supplementary methods and figures. [file 13073_2014_107_MOESM1_ESM.pdf]

Supplementary Methods 2
Microbiome processing and feature extraction 2
Sequencing and reference mapping 2
Patient selection criteria 2
Pruning by correlation 2
Data transformation 2
Calculation of between-subject distances (beta diversity) 2
Immunochip processing and feature extraction 3
NOD2 multiple variant aggregation 3
Immunochip fine mapping and credible set identification 3
Association testing 3
Clinical covariates 3
Linear tests with arcsine-square root transform 4
Outlier detection and filtering 4
Microbiome-wide comparison across cohorts 4
Imputation of missing clinical data 5
Clinical covariate sensitivity analysis 5

Supplementary Figures 6

Supplementary References 15

Supplementary Methods

Microbiome processing and feature extraction

Sequencing and reference mapping

We extracted 16S ribosomal RNA from mucosal tissue biopsies as described previously [1]. We clustered sequences into operational taxonomic units (OTUs) using the QIIME [2] `pick_otus.py` script with method "uclust_ref" and the `--suppress_new_clusters` option, and all other default settings to map the sequences by references against the 97% similarity reference sequences in the 13_8 Greengenes release [3]. 91.1% of sequences matched the reference set. After mapping to the reference set, the samples contained 14,315,989 sequences, with 28,689.36 ± 29,889.65 sequences per sample (mean ± s.d.). For further analysis we subsampled 2,000 sequences, without replacement, from each sample to control for sequencing effort; this sequence depth is expected to be more than sufficient to measure shifts in dominant taxa [4]. We collapsed OTUs according to the Greengenes reference sequence taxonomy assignments, and carried forward taxon groups at all taxonomic levels.

Patient selection criteria

All patients were diagnosed with Crohn's disease or ulcerative colitis. Patients were between the ages of 18 and 75. For all analyses except the meta-analysis of clinical covariates and *NOD2,* we excluded patients with a history of antibiotic usage in the month preceding sampling. For the small number of patients that had more than one sequenced sample, we selected a non-inflamed biopsy first. In the rare case that there was more than one non-inflamed biopsy from the same general location we collapsed those samples together; in the rare case that these samples were from different general biopsy locations (terminal ileum versus colon) we selected terminal ileum first.

Pruning by correlation

In order to remove redundant signals from the data prior to performing genome-microbiome association tests, we calculated all-against-all Pearson's correlations within taxa and KEGG pathways. We then clustered these correlations using complete linkage at a threshold of 0.95 Pearson's correlation. Within each cluster we selected the taxon/pathway with the highest average abundance as the representative feature. Supplementary Table 6 contains a list of taxa that were clustered during this step.

Data transformation

To reduce heteroscedasticity while preserving zero-nonzero relationships within taxa, we applied the arcsine-square root transform to all taxon relative abundances, as described previously [5]. In subsequent linear association tests we excluded taxa with zero abundance in greater than 25% of samples to avoid the effects of zero-inflated distributions on the linear model. For all analyses we included only those taxa present in at least 10% of samples.

Calculation of between-subject distances (beta diversity)

We calculated principal coordinates of between-sample (beta) diversity for association testing with host genetic loci using the first three axes of principal coordinates analysis (Poi) via the standard R function `cmdscale`. We included several measures of between-

sample diversity. To measure the phylogenetic distance between samples we used weighted UniFrac [6] distances calculated with QIIME and the Greengenes reference phylogeny.

Immunochip processing and feature extraction

NOD2 multiple variant aggregation

For NOD2 tests we extracted six known causal variants described previously [7]: rs2066844 (R702W), rs2066845 (G908R), rs5743277, rs5743293 (fs1007insC), rs104895431 and rs104895467. These variants are almost completely independent, and thus we were able to simply take the sum of risk alleles present at any of the six across the cohorts, and still have only subjects with zero, one, or two total risk alleles. We binned two subjects with 3 total risk alleles across the six variants with the 2-risk allele subjects. We then used these counts in subsequent tests for association with the microbiome. All six variants are location in NOD2 introns.

Immunochip fine mapping and credible set identification

The variants we tested for these genes were the top published variants in the largest GWAS analysis to date [8], or the best proxies by linkage disequilibrium (LD) present on Immunochip; however, as evidenced by our success with fine mapping of additional *NOD2* variants, single variants may not represent the full signal of disease risk or microbiome interaction within their respective loci. In a cohort of 33,938 individuals (partially described elsewhere [8], the remainder unpublished) we used regions of high genotyping density on the Immunochip to identify additional independent signals of disease risk near six other SNPs (Supplementary Table 7). Although apart from *NOD2* incorporating these additional variants had mixed results, we expect that as the number of testable loci grows with cohort sizes, the approach will be essential in identifying genome-microbiome interactions at complex risk loci.

We performed fine mapping using a Bayesian based approach (manuscript in preparation). We constructed a posterior probability function of the independent associations given the observed phenotypes and genotypes. The most probable set of independent associations was identified by maximizing this posterior probability in using a greedy algorithm [9]. Uniform prior and steepest descent approximation were used in the calculations. The credible set of SNPs was defined as the minimal set of SNPs such that their sum of posterior probabilities is greater or equal to 95% [10]. SNPs within the credible set were ranked based on their posterior probabilities.

Association testing

Clinical covariates

In all association tests we included linear covariates for antibiotic usage within the last month, immunosuppressant usage within the last month, biopsy inflammation status, age, at time of sampling, gender, general biopsy location (ileum, colon, or pre-pouch ileum), CD/UC diagnosis, disease location (Montreal classification [11] locations E1, E2, or E3 for UC; L1, L2, or L3 for CD), cohort membership (i.e. Boston vs. Toronto vs. Groningen). We excluded time since diagnosis due to high correlation with age (Pearson's correlation coefficient = 0.487; $p < 2.2\times10^{-16}$). To avoid including redundant clinical covariates in the linear regression, we identified clusters of redundant variables by the amount of information they shared. To determine the level of informational overlap between

covariates, we first recoded each categorical variable using multiple binary dummy variables, and discretized each continuous variable using the infotheo package in R [12]. We then calculated the maximum uncertainty coefficient [13] between each pair of variables. The uncertainty coefficient measures the amount of information in one variable that cannot be explained by another. For any group of variables in which each variable contained at least 75% of the information in all the others, we selected the single variable with highest entropy as a representative for the group. At this level all covariates were retained (Supplementary Figure 7).

Linear tests with arcsine-square root transform

Due to common occurrence of samples with zero relative abundance of a given taxon, taxon relative abundances are not readily amenable to power transform for normalization, therefore we apply the arcsine-square-root transform prior to regression, as done in previous microbiome studies [1, 5, 14]. The arcsine-square root transform applies naturally to values on the unit interval, such as relative abundances. It has the effect of stabilizing variance and reducing heteroscedasticity. It is similar to a log or power transformation in that smaller values are decompressed and larger values are relatively compressed, but it has the additional advantage that it handles zero values by definition. The general form of the model for the relative abundance of a given bacterial taxon in subject *i* regressed on genetic variant *j,* while controlling for various covariates, is therefore:

$$\sin^{-1}\sqrt{y_i} = \beta_0 + \beta_1 A_{ij} + \beta_2 C_{i1} + \beta_3 C_{i2} + \beta_4 C_{i3} + \cdots + \varepsilon_{ij}$$

where A_{ij} is the risk allele count for genetic variant *j* in subject *i* (0, 1, or 2); C_{i1}, C_{i2}, ... are the clinical covariates described in the main text. After applying the arcsine-square root transformation we performed standard multivariate regression in R[15] to test the null hypothesis that coefficient β_1 was equal to zero.

Outlier detection and filtering

In every association test we excluded samples with values of the bacterial taxon relative abundance that exceeded three times the interquartile range beyond the median value, as estimated using the R function `boxplot`.

Microbiome-wide comparison across cohorts

To test for conservation of associations a particular genetic locus with the entire microbiome across cohorts, we first obtained individual p-values and regression coefficients for interaction of that locus with each individual microbiome feature. For a given locus we then selected the subset of microbiome features that were nominally significant ($p < 0.05$) in at least one cohort, and compared the directionality (signs) of the coefficients for corresponding microbiome features from one cohort to the other. We assessed the similarity of associations between cohorts using the phi coefficient, the Pearson's correlation of the positive/negative signs of the coefficients. We used the phi coefficient of the signs of the regression coefficients instead of correlation of the actual regression coefficients because the magnitude of the regression coefficients is highly correlated with the mean of the particular microbiome feature being considered. Using the sign tests for conservation of the directionality of the effect across cohorts. We identified genes whose microbiome associations were conserved between at least two cohorts (FDR < 0.05 when comparing Boston to Toronto or Boston to Netherlands), then constructed a gene interaction network from those using GeneMANIA [16]. The gene network displayed

excludes those genes with no interactions found (LFNG, VWC2). We used GeneMANIA to test for enrichment of functional modules at FDR < 0.05.

Imputation of missing clinical data

Recent antibiotics usage history was not collected for the Groningen cohort; we imputed these values using a Random Forests classifier [17] with default settings in the R randomForest package [18] trained on all taxa, functions, and clinical data for other subjects, with estimated accuracy of 87.3% (96.74% expected accuracy for true "No" subjects, 58.7% error for true "Yes" subjects); two of 135 Groningen subjects were predicted to have had recent administration of antibiotics.

Clinical covariate sensitivity analysis

We verified that effect sizes and directionality were highly conserved regardless of whether pre-pouch ileum samples were included or excluded (Spearman's rho = 0.92; p= 4.0×10^{-6} for bacterial taxa; Supplementary 8) subjects over age 50 were excluded (Spearman's rho = 0.94; p= 4.1×10^{-6}; Supplementary Figure 9). We verified in a subset of 377 samples that the addition of covariates for mesalamine and steroid usage did not alter the directionality of any nominally significant results (directionality was conserved for 6 out of 6 nominally significant taxon associations with *NOD2*).

Supplementary Figures

	Boston	Toronto	Groningen
**Age**			
median ± m.a.d.	38.1 ± 11.3	43.9 ± 15.0	40.75 ± 15.8
**Biopsy Location**			
Colon	111	29	141
Ileum	41	19	21
Pre-pouch Ileum	0	113	0
**Disease**			
CD	95	23	103
UC	57	137	59
**Disease location**			
L1, L2, L3	19, 26, 50	3, 7, 12	26, 28, 49
E1, E2, E3	3, 19, 34	2, 19, 113	6, 14, 39
**Gender**			
M	67	82	78
F	85	78	84
**Immunosuppresants**			
Yes	96	5	112
No	56	155	50
**Inflamed biopsy**			
Yes	48	18	86
No	104	142	76
**Years since diagnosis**			
median ± m.a.d.	11.8 ± 9.1	15.3 ± 10.1	6.4 ± 6.5

Supplementary Figure 1. Summary of clinical covariates by cohort
Real-valued entries (Years since diagnosis, Age) show median +/- median absolute deviation for robustness to outliers. Antibiotics entries include imputed values for Groningen cohort (see Supplementary Methods).

	Colon	Ileum	Pre-pouch Ileum
**Age**			
median ± m.a.d.	39.0 ± 14.1	36.5 ± 13.1	48.4 ± 11.5
**Disease**			
CD	157	64	0
UC	124	16	113
**Disease location**			
L1, L2, L3	27, 56, 73	21, 5, 38	0, 0, 0
E1, E2, E3	9, 35, 79	2, 4, 9	0, 13, 98
**Gender**			
M	131	39	57
F	150	41	56
**Immunosuppresants**			
Yes	165	45	3
No	116	35	110
**Inflamed biopsy**			
Yes	121	28	3
No	160	52	110
**Years since diagnosis**			
median ± m.a.d.	8.5 ± 7.7	9.2 ± 6.7	17.7 ± 10.6

Supplementary Figure 2. Summary of clinical covariates by biopsy location
Real-valued entries (Years since diagnosis, Age) show median +/- median absolute deviation for robustness to outliers. Antibiotics entries include imputed values for Groningen cohort (see Supplementary Methods).

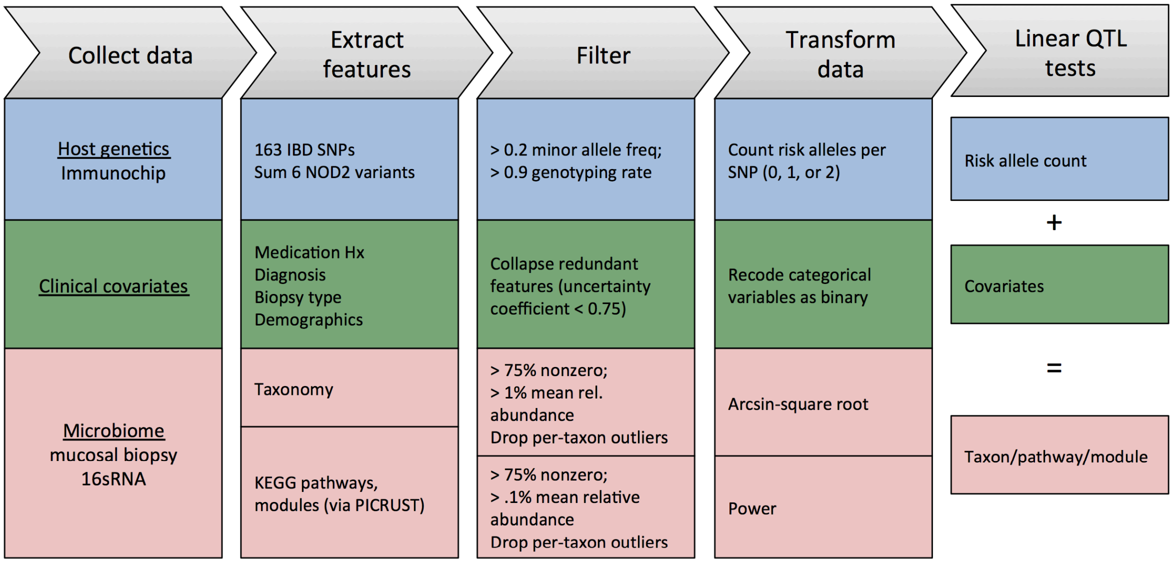

Supplementary Figure 3. Detailed schematic of host genome-microbiome association testing methodology

Host genome-microbiome interaction testing involves potentially thousands or millions of genetic polymorphisms and hundreds or thousands of bacterial taxa and genes. Full feature-by-feature interaction testing is likely to be underpowered in all but the largest cohorts or meta-analyses; therefore our methodology includes careful feature selection from both data types. Raw genetic polymorphisms were derived from Immunochip data and filtered by known IBD associations from large-cohort GWAS studies. NOD2 risk signal was calculated as the sum of 6 independent causal variants as described in the main text. SNPs were further filtered by minor allele frequency and genotyping rate. For each of the 163 previously published IBD-related SNPs, we counted the number of risk alleles for a given subject (0, 1, or 2). Microbiome operational taxonomic units (OTUs) were grouped by lineage at all taxonomic levels using Greengenes. These were filtered by prevalence (rate of presence) and mean relative abundance. Clinical covariates were collapsed when they contained redundant signals (see Methods); categorical variables were recoded as binary dummy variables. Finally, linear tests were performed for association of microbiome features with individual SNPs while controlling for clinical covariates.

a

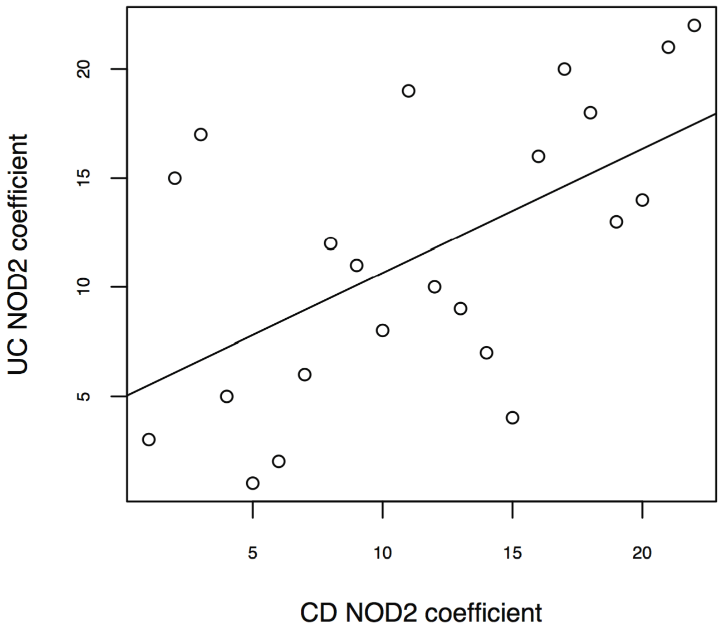

Supplementary Figure 4. Comparison of NOD2 association tests in subjects with UC versus subjects with CD

A value on the horizontal axis represents the (negative log) significance of associations between a bacterial feature and NOD2 including only samples from subjects with CD; a corresponding value on the vertical axis represents the (negative log) significance of the same test when including only subjects with UC. Although the two sets of subjects are completely independent, the two sets of test results are highly correlated. a, taxon-NOD2 coefficients ($p = .006$, Spearman's correlation 0.57).

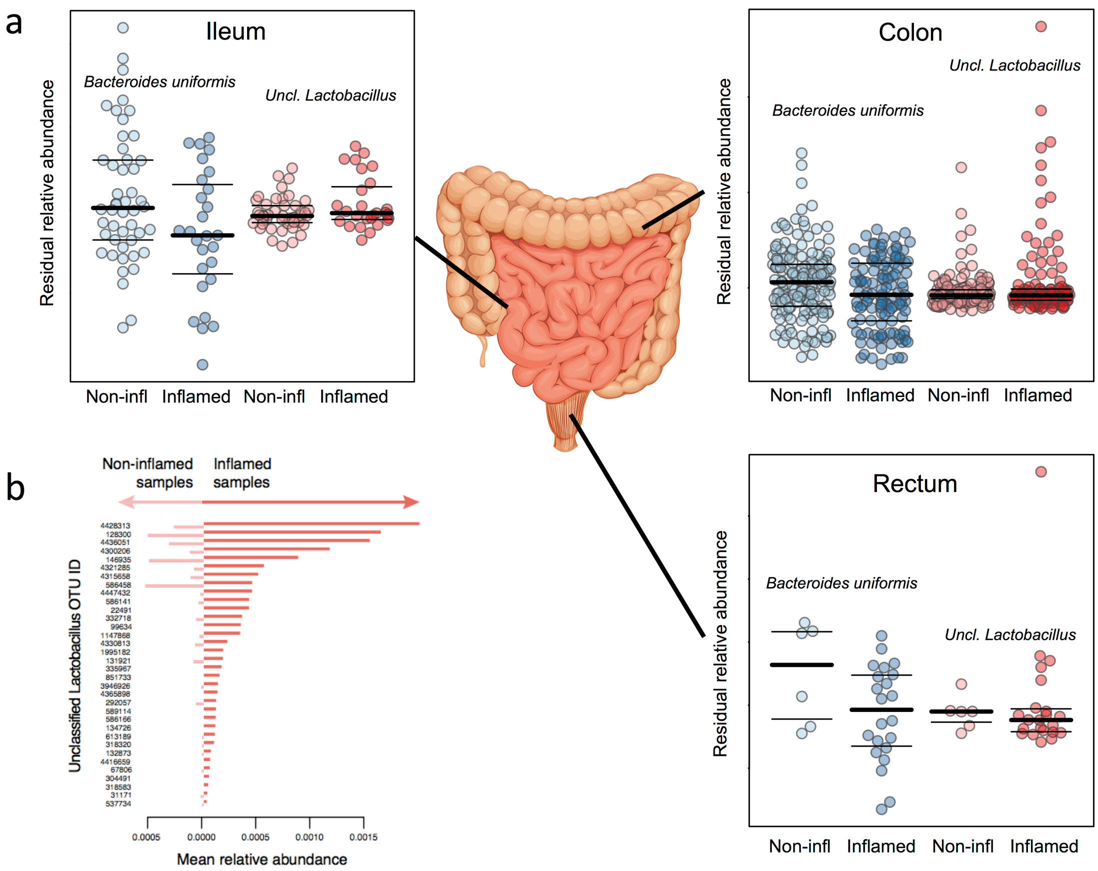

Supplementary Figure 5. Differentially abundant taxa on inflamed biopsies
a, the species *Bacteroides uniformis* is significantly less abundant on inflamed biopsies; a number of unclassified OTUs from the genus *Lactobacillus* are significantly more abundant on inflamed samples. These effects are true at different biopsy locations. b, a plot of independent OTUs assigned to the unclassified *Lactobacillus* taxonomic group, showing consistently higher mean relative abundance in inflamed samples than in non-inflamed samples.

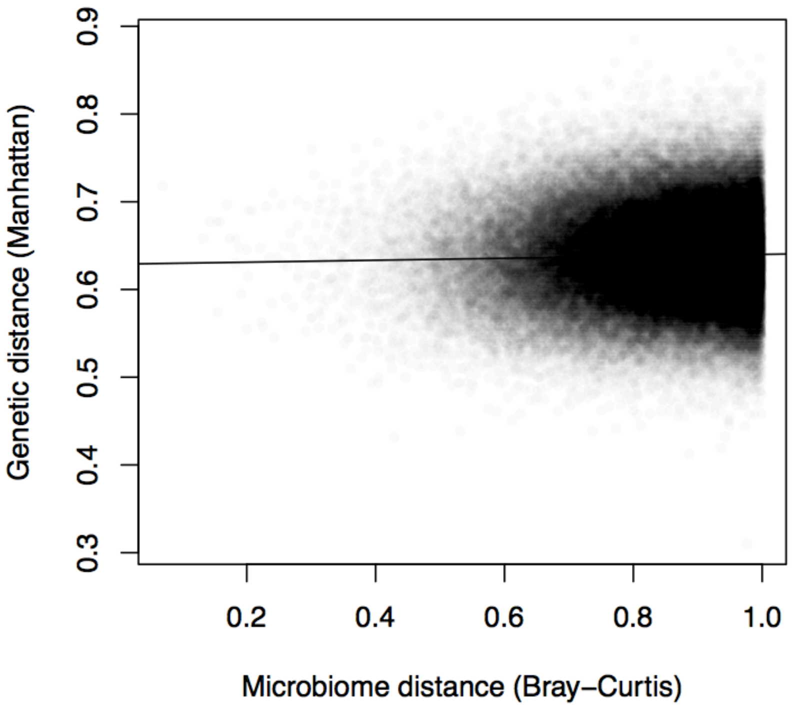

Supplementary Figure 6. Between-individual genomic distance correlates with microbiome distance
Scatterplot of genetic distance between pairs of individuals (Manhattan distance) against distances between microbiomes of those pairs of individuals (Bray Curtis distance of genus-level taxa).

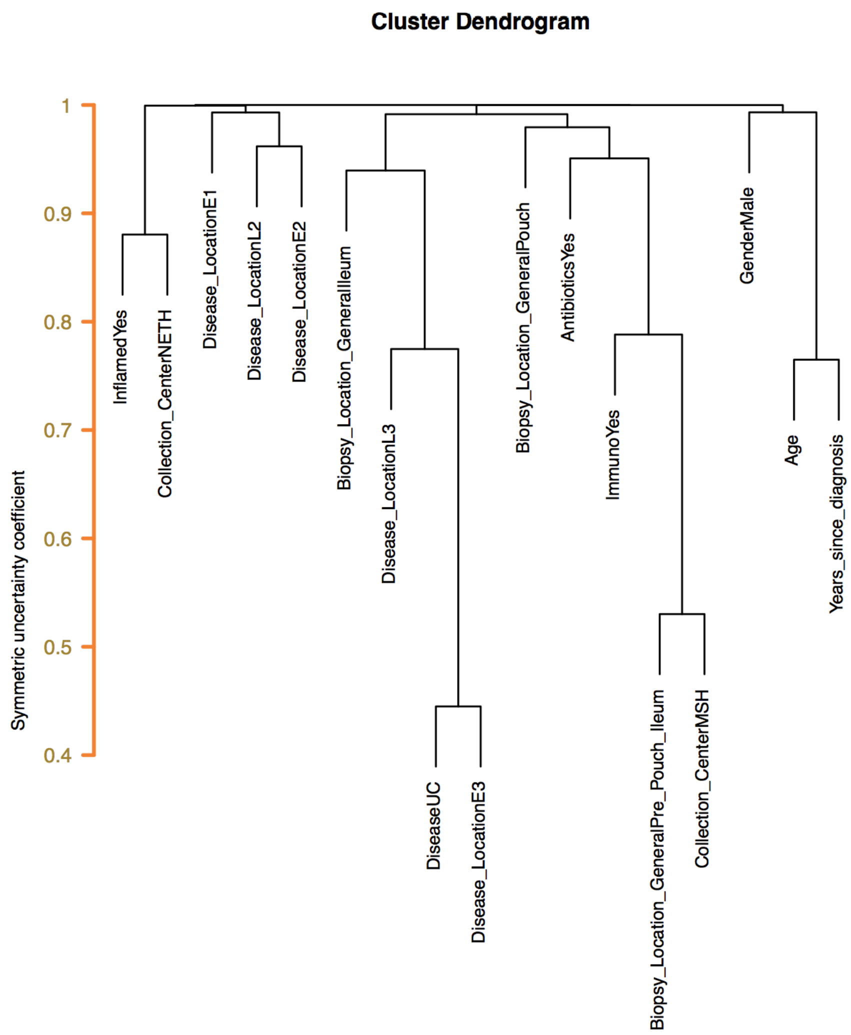

Supplementary Figure 7. Covariate redundancy clustering
Complete-linkage hierarchical clustering of clinical covariates based on pairwise uncertainty coefficient. Lower pairwise uncertainty coefficient indicates higher shared information between features.

a

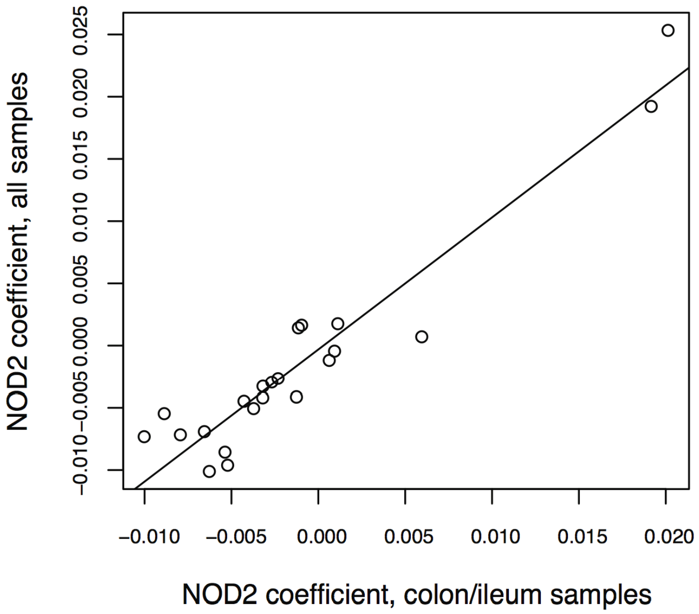

Supplementary Figure 8. Sensitivity analysis of inclusion vs. exclusion of pre-pouch ileum samples in NOD2 association

A value on the horizontal axis represents the (negative log) significance of associations between a bacterial feature and *NOD2* when excluding pre-pouch ileum samples; a corresponding value on the vertical axis represents the (negative log) significance of the same test when including the pre-pouch ileum samples. The two sets of test results are highly correlated. a, taxon-*NOD2* coefficients ($p = 4.0\times10^{-6}$, Spearman's rho = 0.92). The figure is not intended to demonstrate that the effects are the same when tested only in the pre-pouch ileum samples, as that would require many more samples of that type. The figure demonstrates that the directionalities and effect sizes of the NOD2-microbiome associations do not change significantly when we choose between including and excluding the pre-pouch ileum samples.

a

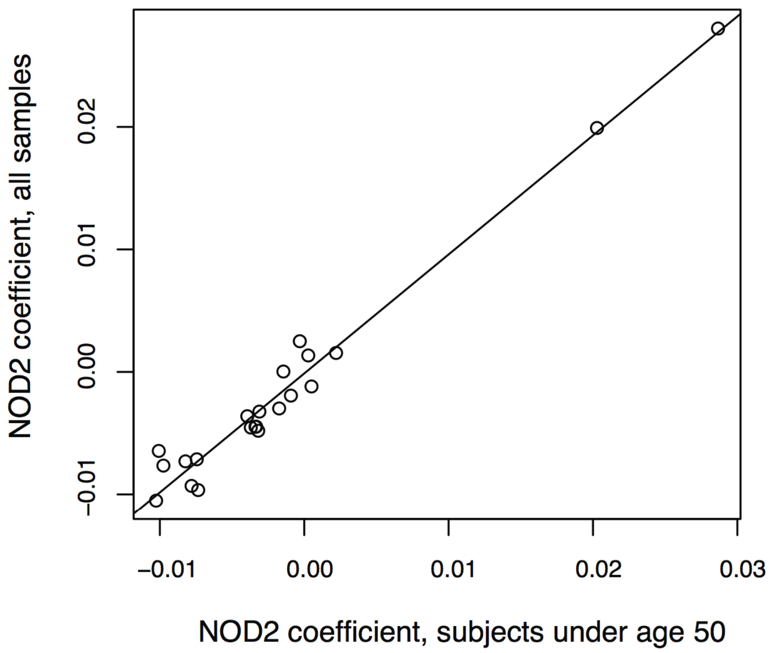

Supplementary Figure 9. Comparison of NOD2 association tests with and without subjects over age 50
A value on the horizontal axis represents the (negative log) significance of associations between a bacterial feature and NOD2 when excluding samples from subjects over age 50; a corresponding value on the vertical axis represents the (negative log) significance of the same test when including all samples. The two sets of test results are highly correlated. a, taxon-NOD2 coefficients ($p = 4.1\times10^{-6}$, Spearman's correlation 0.94); The figure is not intended to demonstrate that the effects are the same when tested only in the over-50 samples, as that would require many more samples of that type. The figure demonstrates that the directionalities and effect sizes of the NOD2-microbiome associations do not change significantly when we choose between including and excluding the over-50 samples.

Supplementary References

1. Huttenhower C, Gevers D, Knight R, Methé BA, Nelson KE, Pop M, Creasy HH, Giglio MG, Petrosino JF, Abubucker S, Badger JH, Others: **A framework for human microbiome research**. *Nature* 2012, **486**:215–221.
2. Caporaso JG, Kuczynski J, Stombaugh J, Bittinger K, Bushman FD, Costello EK, Fierer N, Peña AG, Goodrich JK, Gordon JI, Huttley GA, Kelley ST, Knights D, Koenig JE, Ley RE, Lozupone CA, McDonald D, Muegge BD, Pirrung M, Reeder J, Sevinsky JR, Turnbaugh PJ, Walters WA, Widmann J, Yatsunenko T, Zaneveld J, Knight R: **QIIME allows analysis of high-throughput community sequencing data.** *Nat Methods* 2010, **7**:335–6.
3. McDonald D, Price MN, Goodrich J, Nawrocki EP, DeSantis TZ, Probst A, Andersen GL, Knight R, Hugenholtz P: **An improved Greengenes taxonomy with explicit ranks for ecological and evolutionary analyses of bacteria and archaea.** *ISME J* 2012, **6**:610–8.
4. Kuczynski J, Costello EK, Nemergut DR, Zaneveld J, Lauber CL, Knights D, Koren O, Fierer N, Kelley ST, Ley RE, Gordon JI, Knight R: **Direct sequencing of the human microbiome readily reveals community differences.** *Genome Biol* 2010, **11**:210.
5. Huttenhower C, Gevers D, Knight R, Abubucker S, Badger JH, Chinwalla AT, Creasy HH, Earl AM, Fitzgerald MG, Fulton RS, others: **Structure, function and diversity of the healthy human microbiome**. *Nature* 2012, **486**:207–214.
6. Lozupone C, Knight R: **UniFrac: a New Phylogenetic Method for Comparing Microbial Communities**. *Appl Environ Microbiol* 2005, **71**:8228–8235.
7. Rivas M a, Beaudoin M, Gardet A, Stevens C, Sharma Y, Zhang CK, Boucher G, Ripke S, Ellinghaus D, Burtt N, Fennell T, Kirby A, Latiano A, Goyette P, Green T, Halfvarson J, Haritunians T, Korn JM, Kuruvilla F, Lagacé C, Neale B, Lo KS, Schumm P, Törkvist L, Dubinsky MC, Brant SR, Silverberg MS, Duerr RH, Altshuler D, Gabriel S, et al.: **Deep resequencing of GWAS loci identifies independent rare variants associated with inflammatory bowel disease.** *Nat Genet* 2011, **43**:1066–73.
8. Jostins L, Ripke S, Weersma RK, Duerr RH, McGovern DP, Hui KY, Lee JC, Schumm LP, Sharma Y, Anderson CA, others: **Host-microbe interactions have shaped the genetic architecture of inflammatory bowel disease**. *Nature* 2012, **491**:119–124.
9. Huang H, Chanda P, Alonso A, Bader JS, Arking DE: **Gene-based tests of association.** *PLoS Genet* 2011, **7**:e1002177.
10. Maller JB, McVean G, Byrnes J, Vukcevic D, Palin K, Su Z, Howson JMM, Auton A, Myers S, Morris A, Pirinen M, Brown MA, Burton PR, Caulfield MJ, Compston A, Farrall M, Hall AS, Hattersley AT, Hill AVS, Mathew CG, Pembrey M, Satsangi J, Stratton MR, Worthington J, Craddock N, Hurles M, Ouwehand W, Parkes M, Rahman N, Duncanson A, et al.: **Bayesian refinement of association signals for 14 loci in 3 common diseases.** *Nat Genet* 2012, **44**:1294–301.
11. Satsangi J, Silverberg MS, Vermeire S, Colombel J-F: **The Montreal classification of inflammatory bowel disease: controversies, consensus, and implications.** *Gut* 2006, **55**:749–53.
12. Meyer PE: **infotheo: Information-Theoretic Measures**. 2012.
13. Press WH, Flannery BP, Teukolsky SA, Vetterling WT: *Numerical Recipes in C: The Art of Scientific Computing*. 3rd edition. Cambridge University Press; 1992:761.
14. Morgan XC, Tickle TL, Sokol H, Gevers D, Devaney KL, Ward D V, Reyes JA, Shah SA, LeLeiko N, Snapper SB, Bousvaros A, Korzenik J, Sands BE, Xavier RJ, Huttenhower C: **Dysfunction of the intestinal microbiome in inflammatory bowel disease and treatment.** *Genome Biol* 2012, **13**:R79.

15. R Core Team: **R: A Language and Environment for Statistical Computing**. 2012.
16. Zuberi K, Franz M, Rodriguez H, Montojo J, Lopes CT, Bader GD, Morris Q: **GeneMANIA prediction server 2013 update.** *Nucleic Acids Res* 2013, **41**(Web Server issue):W115–22.
17. Breiman L: **Random Forests**. *Mach Learn* 2001, **45**:5–32.
18. Liaw A, Wiener M: **Classification and Regression by randomForest**. *R News* 2002, **2**:18–22.
